# The Impact of Peri-Operative Nutritional Status on Survival in Gastroesophageal Adenocarcinoma

**DOI:** 10.3390/curroncol32040186

**Published:** 2025-03-21

**Authors:** Gary Tincknell, Tamara Bosward, Karen Fildes, Hayley Batchelor, Bronwyn Freeman, Mouhannad Jaber, Marie Ranson, Jennifer Haughton, Daniel Brungs

**Affiliations:** 1 Illawarra Shoalhaven Local Health District, Wollongong, NSW 2500, Australia; 2 Graduate School of Medicine, University of Wollongong, Wollongong, NSW 2522, Australia; 3 School of Science, University of Wollongong, Wollongong, NSW 2522, Australia; 4 School of Medical Indigenous and Health Science, University of Wollongong, Wollongong, NSW 2522, Australia

**Keywords:** gastric cancer, esophageal cancer, surgery, peri-operative nutrition, overall survival, disease-free survival, malnutrition, patient-generated subjective global assessment

## Abstract

In patients with gastric, gastroesophageal junction or esophageal adenocarcinoma (GOC), peri-operative multimodal therapies have improved survival; however, prognosis remains underwhelming. Pre-operative nutritional decline and weight are linked with poorer patient outcomes. This study retrospectively analyzed the impact of peri-operative nutritional status (as assessed by patient-generated subjective global assessment, PG-SGA), and weight loss on the survival of patients undergoing curative surgery for GOC (2013 to 2022). Of the 148 patients who underwent surgery, PG-SGA and weight data were available for 107 (72%) and 121 (82%), respectively. At presentation, 44% (*n* = 47) of patients were well nourished, dropping to 17% (*n* = 18) post-operatively. Lower post-operative nutritional status correlated to worse overall survival (OS) (*p* < 0.001). Patients who stayed well nourished or improved their nutritional status had better survival outcomes (HR: 2.7; 95%CI: 1.2–6.1; *p* = 0.01). Significant weight loss (>10%) was ubiquitously observed in 54% (*n* = 64) of patients, and this group had shorter OS (HR: 2.2; 95%CI: 1.2–4.1; *p* = 0.009). In conclusion, both nutritional decline and weight loss negatively impacted survival. Maintenance of nutritional status over the peri-operative period resulted in better outcomes. This study highlights the need for improved nutritional support during curative treatment in GOC.

## 1. Introduction

Gastric and esophageal adenocarcinomas are highly aggressive tumors with poor prognosis despite advancements in therapy, with a 5-year overall survival (OS) less than 25% [1]. Gastric and esophageal adenocarcinomas (hereafter termed gastroesophageal cancer, GOC) have the highest prevalence of malnutrition of any malignancy [2], with malnutrition reported in up to 80% of patients [3]. Malnutrition is a deficiency of nutrient intake, characterized by lean muscle wasting, weight loss, and macronutrient and micronutrient deficiencies [4].

Complete surgical resection, usually as part of multimodal therapy incorporating neoadjuvant chemoradiotherapy or peri-operative chemotherapy, is the sole potentially curative treatment in GOC [5,6]. There are multifactorial contributors to malnutrition in early GOC including mechanical obstruction of the digestive tract, adverse treatment side effects, malabsorption, and cancer-associated cachexia [7,8]. Malnutrition has been associated with longer length of hospital stay, adverse surgical outcomes and complications, and increased healthcare costs [9]. Conversely, being well nourished is associated with greater response to chemotherapy and radiotherapy in oncology patients [10]. Malnourishment in GOC patients has been shown to be associated with poorer OS [11,12].

The scored patient-generated subjective global assessment (PG-SGA) is a highly sensitive and specific malnutrition assessment tool considered the gold-standard method to assess nutritional status in oncology patients [2,13]. The PG-SGA captures participant information using four domains: weight history, dietary intake, nutrition-impact symptoms, and functional limitations [14,15]. The healthcare professional next assesses stores of lean muscle, adipose tissue and oedema and identifies disease states and factors impacting nutritional requirements and metabolic demand [14,15]. The PG-SGA has been shown to be significantly associated with clinical outcomes in a number of trials in GOC [16,17].

Malnutrition at cancer diagnosis is recognized as an important prognostic factor. Unintentional weight loss of 5% over the 6–12 months prior to diagnosis has been correlated with poorer prognosis in various tumors [18,19]. Patients presenting with a low body mass index (BMI) have shorter overall and disease-free survival, and those with higher presenting BMI have similar or improved survival [20]. The impacts of weight loss during peri-operative treatments are less certain. A retrospective study of GOC patients showed that a weight loss of 5% over the neoadjuvant period was associated with poorer OS [21]. In advanced, unresectable GOCs, a weight loss of just 3% from baseline during the first treatment cycle was enough to predict poorer OS across three pooled clinical trials, although prognostic benefit was not seen in patients under treatment in first-line clinical trials [22].

This study aimed to determine the impact of nutritional status and weight change on the survival of patients with GOC undergoing curative surgery.

## 2. Materials and Methods

This retrospective review included all patients with esophageal, gastroesophageal junction and gastric adenocarcinomas who were treated with curative surgery within the Illawarra Shoalhaven Local Health District (ISLHD), New South Wales, Australia. Patients were treated between August 2013 and September 2022. Histological types other than adenocarcinoma were excluded, but adenocarcinoma subgroups (i.e., signet ring) were included in the analysis. All patients were 18 years or over. Patients received treatment consisting of either upfront surgery, neoadjuvant chemoradiotherapy or peri-operative chemotherapy followed by surgery as per their treating oncologists. Patients were recommended nutritional supplementations and prescribed enteral feeds as per treating dietitians and clinicians. GOC patients treated at ISLHD can be assessed in a multidisciplinary clinic consisting of experienced oncology dietitians and a specialist GOC clinical nurse consultant, particularly those patients undergoing multimodality therapy. This research was approved by the South Western Sydney Local Health District HREC via a waiver of consent (N^o^:HREC/15/LPOOL/121).

Patients were identified from ISLHD electronic medical records and the NSW Cancer Registry. Staging assessments were performed according to the American Joint Committee on Cancer Tumor, Node and Metastasis (TNM) staging system, version 8 [23]. As a subset of patients received neoadjuvant treatments, tumor stage at diagnosis was determined by the clinical TNM stage, unless the pathological TNM stage was higher.

PG-SGA and weight assessments occurred at two time points, at diagnosis and 1–3 months post-operatively. The PG-SGA was completed by an experienced dietitian. The PG-SGA determined nutritional status by three categories: A (well nourished), B (moderate/suspected malnutrition) or C (severely malnourished) [15]. It was hypothesized that patients who maintained or improved their nutritional status would have improved survival compared to those patients who remained malnourished or became malnourished. Patients categorized as “Nourished/Improved” maintained their PG-SGA A status post-operatively or improved their level from PG-SGA C to PG-SGA B or A, or from PG-SGA B to A. Patients whose PG-SGA worsened from PG-SGA A to PG-SGA B or C, or remained PG-SGA B or C, were classified as “Deteriorated/Malnourished”.

### 2.1. Outcomes

The primary outcome of interest is patient OS in relation to nutritional status. Secondary outcomes include disease-free survival, changes in weight, changes in nutritional assessment score and their impact on survival.

### 2.2. Statistics

Patient clinicopathological variables were compared by Chi-squared test for categorical variables and one-way ANOVA tests for continuous variables. Intrapatient comparisons at different time points were compared using paired *t*-tests. Overall survival (OS) was determined from date of diagnosis to death or censored at date of last medical contact. Disease-free survival (DFS) was determined from date of diagnosis to date of disease recurrence. The Kaplan–Meier method and log-rank tests were used. Univariate and multivariate analysis were performed by Cox proportional hazards models to assess patient survival. Variables with a univariate *p* < 0.1 were assessed in the multivariate analysis.

Statistical analysis was performed with R version 4.2.1 [24].

## 3. Results

### 3.1. Demographics

One hundred and forty-eight patients underwent curative intent surgery for GOC between 2013 and 2022, with patient demographics summarized in Table 1. The majority were male (*n* = 109, 73.6%) with a median age 69.1 years (range 40–89 years). Eighty-eight participants (59.5%) underwent neoadjuvant treatments, with either peri-operative chemotherapy (*n* = 25) or neoadjuvant chemoradiotherapy (*n* = 63). Of those patients undergoing peri-operative chemotherapy, 19 (76%) received the FLOT protocol [25] and 6 (24%) received the MAGIC protocol [26]. All patients treated with chemoradiotherapy received the CROSS protocol [27]. In total, 89% (*n* = 78) of the patients undergoing pre-operative therapies had their PG-SGA recorded at presentation, compared to only 48% (*n* = 29) of patients undergoing upfront surgery.

### 3.2. Nutritional Status

**Figure 1:**
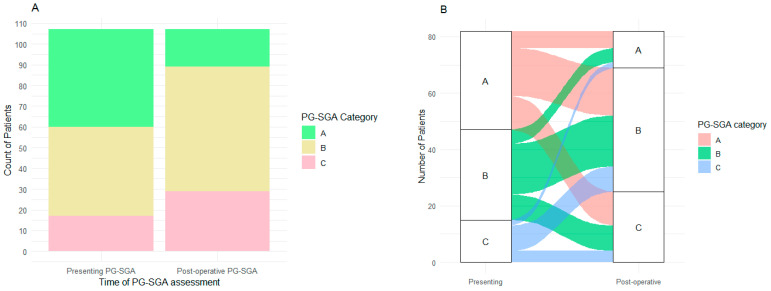
(**A**) The patient-generated subjective global assessment (PG-SGA) status of patients (*n* = 107) at presentation and post-operatively. More patients had poorer nutritional status post-operatively (*p* < 0.001). (**B**) Paired presenting and post-operative PG-SGA of patients (*n* = 82). The figure demonstrates the transition of patients between the PG-SGA groups, with more patients becoming more malnourished post-operatively overall.

The PG-SGA was documented in 107 patients pre- and post-operatively (72%, Figure 1A). Patients had significantly poorer nutritional status post-operatively (*p* < 0.001). Pre-operatively, 47 (44%) were considered well nourished and 60 (56%) were considered malnourished (PG-SGA B 43, PG-SGA C 17). Post-operatively, only 18 (17%) patients were well nourished, with the rest considered malnourished (PG-SGA B 60, PG-SGA C 29; Figure 1A).

**Figure 2:**
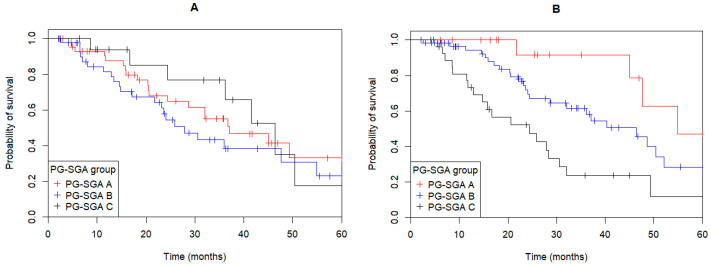
Overall survival (OS) of gastroesophageal adenocarcinoma patients depending on their nutritional status as measured by the patient-generated subjective global assessment (PG-SGA) (**A**) There was no significant difference in OS between PG-SGA nutritional status groups at presentation (*p* = 0.4). (**B**) Post-operative nutritional status was significantly associated with patient OS (*p* < 0.001).

While the nutritional status as measured by PG-SGA at presentation was not associated with OS (*p* = 0.4, Figure 2A), the post-operative nutritional status of patients was significantly associated with OS (*p* < 0.001, Figure 2B). Compared to PG-SGA A, patients with post-operative PG-SGA C had a significantly poorer OS (HR: 5.67; 95%CI: 1.91–16.86; *p* = 0.002), while there was a non-significant trend for patients in the PG-SGA B group (HR: 2.42; 95%CI: 0.84–7.00, *p* = 0.10). Nutritional status by PG-SGA assessment was not significantly associated with DFS at either presentation (*p* = 0.051) or post-operative time points (*p* = 0.24).

**Figure 3:**
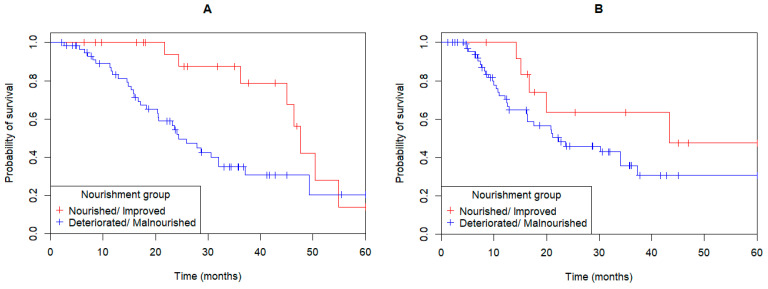
Gastric, gastroesophageal junction and esophageal adenocarcinoma patients who maintained a well-nourished state or improved their severity of malnutrition according to patient-generated subjective global assessment (PG-SGA) category (PG-SGA C to PG-SGA B or A, or PG-SGA B to A), had improved both (**A**) overall survival (HR: 2.71; 95%CI: 1.24–5.95, *p* = 0.007) and (**B**) disease-free survival (HR: 2.68; 95%CI: 1.17–6.14, *p* = 0.01).

Nutritional status was assessed for 82 (77%) patients with the PG-SGA at both presentation and post-operatively (Figure 1B). Those patients who maintained a well-nourished state (PG-SGA A) or improved their nutritional status (PG-SGA C to PG-SGA B or A, or PG-SGA B to A; *n* = 22) had an improved OS compared to those whose nutritional status deteriorated (PG-SGA A to PG-SGA B or C) or remained malnourished (PG-SGA B or C) (median OS 47.6 months vs. 24.4 months; HR: 2.71; 95%CI: 1.24–5.95, *p* = 0.007; Figure 3A). DFS was also significantly improved in patients who maintained a well-nourished state or improved their nutritional status (median DFS: 20.9 months vs. NR; HR: 2.68; 95%CI: 1.17–6.14, *p* = 0.01; Figure 3B) compared to patients whose malnourished state remained static or deteriorated.

### 3.3. Weight Loss

Weight data at both time points were available for 121 (82%) patients. Patients lost a median 10.4% of their presenting weight (range +13% to −27.4%). Almost all participants lost more than 5% of their presenting body weight compared to their post-operative weight (*n* = 106, 87.6%; Table 2), and more than half the patients lost 10% of their presenting weight (*n* = 65, 53.7%; Table 2). Patients who underwent pre-operative treatments had significantly more weight loss than patients having upfront surgery (11.1% vs. 9.7%, *p* < 0.001; Table 2). Greater median weight loss was observed in patients undergoing neoadjuvant chemoradiotherapy (11.9%) than those receiving neoadjuvant chemotherapy (8.6%) (*p* < 0.01). Esophageal and gastroesophageal junction adenocarcinoma patients lost more weight (median −11.3%) in comparison to gastric adenocarcinoma patients (median −8.2%, *p* < 0.001). More advanced tumor stage was associated with degree of weight loss (stage III vs. stage I/II, median −10.4 (range +27.4% to −13%) vs. −10.3% (range +24.7% to −3.3%), *p* < 0.001). Age (*p* = 0.8), ECOG (*p* = 0.1), perineural invasion (*p* = 0.2), and lymphovascular invasion (*p* = 0.7) were not associated with weight change during this same period.

**Figure 4:**
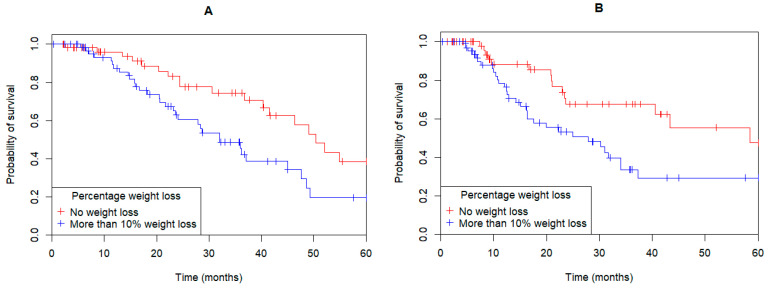
Gastric, gastroesophageal junction and esophageal adenocarcinoma patients with more than 10% percent loss of their presenting weight to their post-operative weight were significantly associated with a poorer overall survival ((**A**) HR: 2.07; 95%CI: 1.15–3.72; *p* = 0.01) and disease-free survival ((**B**) HR: 2.2; 95%CI: 1.19–4.08; *p* = 0.009).

More than half of the patients (*n* = 65, 54%) lost more than 10% of their presenting body weight between presentation and the post-operative period. Loss of greater than 10% of presenting body weight was significantly associated with a poorer OS (median OS 32 months vs. 50.4 months; HR: 2.07; 95%CI: 1.15–3.72; *p* = 0.01; Figure 4A) and DFS (median DFS: 27.9 vs. 58.5 months; HR: 2.2; 95%CI: 1.19–4.08; *p* = 0.009; Figure 4B).

### 3.4. Univariate and Multivariate Models

The strongest association of OS and GOC nutritional status was pre- to post-operative assessment of PG-SGA, categorizing patients into “Nourished/Improved” and “Deteriorating/Malnourished” groups (Figure 3A, *p* = 0.007). In the univariate analysis, pre- to post-operative PG-SGA (*p* = 0.007), stage (*p* = 0.005), and tumor location (*p* = 0.02) were associated with patient OS (Table 3). In the multivariate analysis, pre- to post-operative PG-SGA (*p* = 0.03), gender (*p* = 0.02) and stage (*p* = 0.008) remained significant (Table 3).

## 4. Discussion

This study highlights the significant challenges posed by malnutrition in the treatment of surgically resectable GOCs.

Firstly, we illustrate the ubiquitous nature of malnutrition in GOC, alongside the deleterious impact of curative treatments. Malnutrition was seen in the majority of patients at presentation (56%); most patients became more malnourished during the treatment course (83%), with only a small proportion (17%) remaining well nourished. Similarly, weight loss was almost universal in our cohort, with 88% of patients losing at least 5% body weight and 54% losing at least 10% body weight. The degree of weight loss reported in this study only pertains to changes from the time of presentation to 1–3 months post-operatively. It does not encompass/account for weight lost prior to presentation and does not therefore reflect total loss of usual body weight. However, the application of the PG SGA at the time of presentation does account for reported weight loss in the one to six months prior to presentation (one-month data, where available, is used in preference to six-month data) and therefore reflects weight change over a longer period. Our results are similar to other studies in this population [8,28].

The etiology of malnutrition in GOC cancer is multifactorial. Direct tumor obstruction of the upper gastrointestinal tract is associated with dysphagia, nausea, vomiting and early satiety, ultimately leading to reduced oral intake [29]. Meanwhile, malignancy independently increases nutritional requirements and produces a pro-catabolic state of inflammation which alters metabolism of nutrients required to maintain weight and cellular function [30]. Chemotherapy and radiotherapy negatively affect oral intake due to mucositis, nausea, vomiting and altered taste [31], highlighted by the higher rates of weight loss seen in patients who received neoadjuvant treatments in our cohort. Systemic treatments and surgery produce a catabolic effect in patients, increasing the nutritional requirements of patients which they struggle to meet [32,33]. Surgical resection of esophageal and gastric tumors typically involves dietary texture modification during the post-operative recovery period. Texture-modified diets, such as pureed and minced–moist food, are independently associated with reduced energy intake [34]. Combined with the anatomical changes in the GIT that occur post-operatively that can alter both ingestion (e.g., early satiety, anastomotic strictures) and digestion and absorption (e.g., dumping syndrome), the nutrition support needs of these patients are complex and changeable. Esophagectomy is a particularly invasive surgery with a reported incidence of 42–50% experiencing post-operative complications [35]. Poor nutritional status is independently associated with the increased incidence and severity of post-operative esophagectomy complications [36].

Secondly, we demonstrate the critical impact that malnutrition and weight loss have on patient outcomes. Severe malnutrition (PG-SGA C) in the post-operative period, failure to improve nutritional status (in terms of PG-SGA category), loss of a well-nourished state, and >10% weight loss with treatment were all significantly associated with poorer OS. Moreover, the impacts of these indicators were clinically meaningful. Participants who were well nourished in the post-operative period survived on average 2 years longer than those who were malnourished. Our results agree with other studies which have demonstrated that GOC patients with malnutrition have increased mortality [16,37]. There are a number of postulated reasons for these findings. Malnutrition is known to adversely affect the effectiveness of chemotherapy and radiotherapy [17]. Poor nutritional status is associated with increased incidence of post-operative complications and length of hospital stay [3]. Importantly, an improved OS was associated with those patients who improved their PG-SGA status or maintained a well-nourished state. Despite the significant nutritional challenges faced by this population, these results demonstrate that amelioration of nutrition risk and improvements in nutritional state can have significant clinical benefit in terms of OS, even if malnutrition cannot be eliminated [38]. These results demonstrate the essential importance of early assessment and regular review of patients across the continuum of care by a multidisciplinary team which must include dietitian support.

Thirdly, we highlight the considerable challenges in addressing malnutrition in patients with GOC receiving curative treatment. We identify risk factors associated with weight loss including tumor location and pre-operative treatments, particularly chemoradiotherapy. Other factors in our cohort associated with GOC patient survival include non-modifiable factors including patient gender and tumor stage.

Nutrition support and individualized nutrition counseling is an essential element of patient care during curative treatment and is far broader than just the prescription of oral nutritional supplements or enteral nutrition. Early interdisciplinary support care has been demonstrated in a randomized phase III clinical trial to improve overall survival in metastatic esophageal cancer [38]. Despite multidisciplinary review, weight loss and development of malnutrition were common in our cohort. It is important to highlight that key unmeasured patient factors, such as significant dysphagia, peri-operative complications, delays in the initiation of enteral support due to challenges establishing enteral access, or socioeconomic factors, all need to be considered when considering the impact of the provision of nutrition support. It was beyond the scope of this retrospective study to evaluate the impact of oral nutritional supplements or enteral feeding. More work needs to be undertaken to develop further strategies to prevent nutritional decline. Certainly, the use of pre-operative nutritional supports, including nutritional counseling, ERAS protocols, immunonutrition, and enteral and parenteral feeding, has been demonstrated to reduce peri-operative complications and improve long-term patient outcomes, even if a direct survival measurement has not yet been demonstrated in prospective trials [39,40]. Nutritional supplementation interventions assist with weight loss and sarcopenia prevention and in turn result in reduced peri-operative morbidity and long-term mortality and improved patient quality of life [41,42,43]. The choice of enteral or parenteral supplementation will largely be selected based on unmodifiable patient factors, such as dysphagia, with enteral feeding generally preferred where possible [8,39]. Recent studies in advanced gastrointestinal malignancies suggest that pharmacotherapy with olanzapine may assist in reducing anorexia associated with malignancy, thereby improving appetite and weight [44], although the impact of this approach on survival remains uncertain. With expert multidisciplinary intervention, nutritional status is a potentially modifiable factor, and optimization is essential for improved patient outcomes.

### Limitations of Study

This cohort was a relatively small sample size from a single hospital in Australia, which may impact the transferability of findings to other populations. The retrospective nature of this study will lead to unequal distribution of important confounders between groups, which is likely to have an impact on the results; for example, baseline malnourishment may be overrepresented, or there may be post-operative complications which have not been reported. Documentation of PG-SGA and weight changes were only identified in 72% and 82% of cases, respectively, introducing the possibility of selection bias. Data regarding PG-SGA and weight were only available at two time points in this study. Including more frequent monitoring and intermediate assessment could provide a more dynamic view of the evolving nutritional status throughout treatment and potentially provide additional prognostic information. Data regarding major surgical outcomes and complications, such as anastomotic leaks, which is likely to be significantly influenced by nutritional status, were not available.

The PG-SGA, by definition, is patient-generated. This introduces complications with potentially inadequate or inaccurate completion, such as the under-reporting or over-reporting of symptoms. Patient compliance with prescription of nutritional supplements or dietetic nutritional recommendations could not be assessed in this retrospective analysis. Additionally, extrapolation to culturally and linguistically diverse patients should be interpreted with caution. Post-operative nutritional intervention was not analyzed in this study.

## 5. Conclusions

The nutritional status of patients with GOCs is a vital, potentially modifiable, patient attribute associated with improved survival. Maintenance or improvement of nutritional status was associated with improved overall survival and should be the goal of early intervention. In addition, loss of more than 10% of presenting weight is adversely associated with patient survival. Prevention of nutritional decline needs to be the goal from presentation, and prospective studies are needed to assess the impact of potentially adjustable nutritional variables on the overall survival of patients.

## Figures and Tables

**Table 1:** Participant demographics and cancer characteristics (*n* = 148).

Variable	Total Population
Number (*n*)	148
Age: Median (range, years)	69.1 (40, 89)
Male (*n*, %)	109 (73.6)
ECOG (*n*, %)	
0	82 (61.7)
1	43 (34.6)
2 or 3	5 (3.8)
Tumor location	
Esophagus	46 (31.1)
GOJ	46 (31.1)
Gastric	56 (37.8)
Tumor stage	
I	17 (11.5)
II	41 (27.7)
III	76 (51.4)
IV	14 (9.5)
Tumor Differentiation (*n*, %)	
Well	20 (15)
Moderate	58 (43.6)
Poor	55 (41.4)
Pre-operative regimen (*n*, %)	
Chemotherapy	25 (16.9)
Chemoradiotherapy	63 (42.5)
Nil	60 (40.5)
Presenting PG-SGA (*n*, %)	
A: well nourished	47 (32)
B: moderate/suspected malnutrition	43 (29)
C: Severely malnourished	17 (11)
Not reported	41 (28)

*n*: number of participants; ECOG: Eastern Cooperative Oncology Group; GOJ: gastroesophageal junction; PG-SGA: patient-generated subjective global assessment.

**Table 2:** Average percentage weight change of GOC patients from presentation to post-operative assessment.

	Total Patients (*n* = 121)	Upfront Surgery (*n* = 41)	Pre-Operative Treatment (*n* = 80)	*p* Value
% weight change (median, range)	−10.4 (+13.0, −27.4)	−9.7 (+13, −25.7)	−11.1 (+2.8, −27.4)	<0.001
Number more than 5% weight loss (%)	106 (87.6)	33 (80.5)	73 (91.3)	<0.001
Number more than 10% weight loss (%)	65 (53.7)	19 (46.3)	46 (57.5)	0.039

**Table 3:** Univariate and multivariate analysis of GOC patient nourishment status and OS.

Variable	Univariate OS			Multivariate OS		
	HR	95% Confidence Interval	*p*-Value	HR	95% Confidence Interval	*p*-Value
PG-SGA “Nourished/Improved” vs. “Deteriorated/Malnourished”	2.71	1.23–5.94	0.007	2.81	1.15–6.83	0.03
Age categorical <65 vs. >65	1.24	0.73–2.12	0.42			
Gender Female vs. Male	1.66	0.91–3.02	0.08	1.98	0.55–3.44	0.02
Stage			0.005			0.008
I vs. II	0.63	0.22–1.82		1.72	0.34–8.69	
I vs. III	1.60	0.67–3.78		3.49	0.78–15.59	
I vs. IV	3.21	1.13–9.06		10.09	1.89–53.70	
Tumor location (ref: gastric)			0.02			
GOJ	1.45	0.77–2.72				
Esophagus	1.73	0.93–3.21				
Presenting ECOG performance status (ref: 0)			0.09			
1	1.63	0.94–2.83				
2 or 3	3.07	0.92–10.28				
Neoadjuvant treatment	1.54	0.90–2.66	0.11			

HR: Hazard Ratio; OS: overall survival; PG-SGA: patient-generated subjective global assessment; ref: reference group; GOJ: gastroesophageal junction; ECOG: Eastern Cooperative Oncology Group.

## Data Availability

The data presented in this study are available on request from the corresponding author.

## Abbreviations

The following abbreviations are used in this manuscript:

GOC	gastric, gastroesophageal junction and esophageal adenocarcinoma
PG-SGA	patient-generated subjective global assessment
OS	overall survival
DFS	disease-free survival
